# Telepractice and Dysphagia Management: The Era of COVID-19 and Beyond

**DOI:** 10.1007/s00455-022-10444-2

**Published:** 2022-04-15

**Authors:** Elizabeth C. Ward, Madeline Raatz, Jeanne Marshall, Laurelie R. Wishart, Clare L. Burns

**Affiliations:** 1 Centre for Functioning and Health Research (CFAHR), Metro South Hospital and Health Service, PO Box 6053, Buranda, QLD 4102 Australia; 2 School of Health & Rehabilitation Sciences, The University of Queensland, Brisbane, QLD Australia; 3 Speech Pathology Department, Queensland Children’s Hospital, Brisbane, QLD Australia; 4 Speech Pathology Department, Royal Brisbane & Women’s Hospital, Metro North Hospital and Health Service, Brisbane, QLD Australia

**Keywords:** Telepractice, Speech language pathology, Dysphagia, Feeding disorders

## Abstract

The COVID-19 pandemic drove rapid and widespread uptake of telepractice across all aspects of healthcare. The delivery of dysphagia care was no exception, with telepractice recognized as a service modality that could support social distancing/infection control, overcome service delivery challenges created by lockdowns/service closures, and address consumer concerns about attending in-person appointments. Now, almost two years since most services first rapidly deployed telepractice, it is time to reflect on the big picture, and consider how telepractice will continue as a service option that is sustained and integrated into mainstream dysphagia care. It is also timely to consider the research agenda needed to support this goal. To this end, in this paper we present 4 discussion topics, which raise key considerations for the current and future use of telepractice within adult and pediatric dysphagia services. These are (1) Dysphagia services must meet consumer and service needs; (2) Aspects of dysphagia services can be safely and reliably provided via telepractice; (3) Telepractice can be used in flexible ways to support the delivery of dysphagia services; and (4) Providing quality dysphagia services via telepractice requires planned implementation and evaluation. Then directions for future research are discussed. These considerations are presented to help shift perspectives away from viewing telepractice as simply a COVID-19 “interim-care solution”. Rather, we encourage clinicians, services, and researchers to embrace a future of “integrated care”, where traditional dysphagia services are combined with telepractice models, to enhance the quality of care provided to our clients.

## Introduction

Prior to the COVID-19 pandemic, the exact number of speech language pathologists (SLPs) offering telepractice services across all practice areas was unknown. However, the numbers were considered to be low [1–3], and only a slow, gradual pattern of growth was being seen over time [4]. Specifically in relation to adult and pediatric dysphagia care, very few clinicians were providing services via telepractice [1, 2, 5]. However, the global COVID-19 pandemic significantly changed this, and a rapid increase in the uptake of telepractice by SLP services has been observed across the world [6–13]. Where previously the use of telepractice had been carefully considered and then systematically implemented in discrete settings with recognized need (e.g., services supporting patients in rural and regional areas), with the onset of COVID-19 position papers called for all organizations to consider their telepractice capabilities, and where possible, transition to care via telepractice to reduce transmission risk and continue service delivery [14, 15].

Naturally, this period of transition came with many challenges. In papers which examined SLP experiences with telepractice during COVID, it has been highlighted that some services simply did not have the infrastructure to support telepractice [6, 7, 13]. In addition, many clinicians were unaware of the current evidence for telepractice delivery of dysphagia care, there was a recognized lack of prior training or experience in delivering services via telepractice, a lack of knowledge regarding how to adequately prepare clients for telepractice sessions and manage key issues such as data safety and security, and some faced challenges created by limited reimbursement options [6–13, 16–18]. There was equally a critical absence of general knowledge among allied health professionals about how to operate a telepractice service [16, 18]. Hence, it was a time of great confusion and anxiety for services, clinical staff, and clients. In no prior era had such massive change in health service delivery methods been thrust upon a global population of healthcare workers and their consumers, in such a short period of time. It was not unexpected then that among the many stories of success and positive consumer feedback, there were also stories of service failure and negative staff and client reactions.

The transition to delivering aspects of dysphagia care via telepractice was no exception to this upheaval. Although dysphagia was recognized as a prevalent sequela of COVID-19 [19] there were suddenly disruptions and barriers to providing usual dysphagia management for both COVID and non-COVID patients across every clinical domain. These barriers were created by infection control risks, particularly those associated with clinical and instrumental assessments being classified as aerosol generating procedures [14, 20–22], as well as concerns for high-risk clinical populations [6] such as those in aged care [23] and people with head and neck cancer [HNC] [24, 25]. The situation was then further exacerbated for some by other operational challenges, such as the need to ensure staff had adequate access to necessary personal protective equipment (PPE) [6, 22], and in Australia a barium shortage further impacted the ability to conduct videofluoroscopic assessment (VFSS) services.

To meet service needs, telepractice models to support clinical evaluations of dysphagia were rapidly deployed across a range of healthcare services. In critical care settings, this may have involved remote SLPs guiding nurse-led dysphagia screening via videoconferencing. In outpatient and community/residential age care, this may have involved conducting clinical swallowing examinations and therapy reviews via telepractice into patient homes, alternate facilities (e.g., smaller hospitals with no SLP services), or aged-care homes to triage care. While some of these models (e.g., assessments within critical care) will have less relevance outside an infection control scenario such as COVID-19, others were found to be highly beneficial and had clear benefit for supporting dysphagia care in the post-COVID-19 era. From this experience, there are now a wide range of clinical settings that are re-examining telepractice and the role it can play in the future of dysphagia services.

As such, it is now timely to distill the benefits from the challenges of the past 2 years, and consider the future of dysphagia services and how telepractice will remain part of this. To this end, this paper proposes 4 key discussion topics developed from the experiences of the author group, who are all telepractice clinicians and who were actively involved in supporting clinical implementation, global training, local and national policy change, and research into telepractice during the pandemic. These 4 discussion topics were seen as key considerations to support the sustained uptake of telepractice as part of the future of dysphagia care, where telepractice and in-person care are used when they are most appropriate for both the clinical situation and client need. The 4 topics include the following: (1) Dysphagia services must meet consumer and service needs; (2) Aspects of dysphagia services can be safely and reliably provided via telepractice; (3) Telepractice can be used in flexible ways to support the delivery of dysphagia services; and (4) Providing quality dysphagia services via telepractice requires planned implementation and evaluation. Following these, key directions for future research are discussed.

## Dysphagia Services Must Meet Consumer and Service Needs

Prior to the pandemic, the dominant model of care for dysphagia intervention was in-person care, where the patient with dysphagia attends the SLP’s clinic for all assessment and management appointments. While this may still be “traditional care” for many, it is now recognized that this model is not always optimal for our clients, and may not always be the most efficient and effective way to deliver care. In this modern era where increasing resource demands are driving clinical efficiencies, and where there is greater awareness of the consumer burden associated with accessing care, it is time to re-assess the “status-quo” and consider changing how services are delivered.

It has long been recognized that consumers living in regional and rural areas have increased challenges accessing services, and due to the lack of local services, many experience delays to receiving care. In a recent series of papers describing the implementation of a telepractice model for delivering clinical swallow examinations (CSEs) across 18 regional/rural clinical sites [26, 27], the delay between referral and assessment in the traditional in-person model of care ranged from 2 to 13 days [27]. Clinicians were also expected to travel significant distances to provide care [26, 27]. The issue of clinician travel has also been noted to create unique challenges for clinicians providing home-visiting pediatric feeding services. Similar to the issues described for adult services, traveling long distances to provide pediatric feeding services may lead to clinician fatigue and reduced service availability [28].

For individuals who require access to specialist dysphagia services, barriers to care can be even more significant. Specialist dysphagia services (e.g., HNC services, pediatric feeding care) are often provided by multidisciplinary teams located in larger metropolitan centers. Individuals living outside metropolitan areas are often unable to access such specialist care locally and are required to travel large distances to access services. These challenges were highlighted in recent research that examined the experiences of families accessing a specialist pediatric feeding service for their child’s feeding care [29]. Families reported numerous challenges associated with service access, and 85% reported that attending their appointment took at least half a day. The findings were further reinforced by a second study by Raatz et al. [30] where even though all participants lived ≤ 40 km from the healthcare facility, most families still needed to take at least half a day away from usual activities to attend their in-person appointments. Attending multiple dysphagia appointments may also adversely impact work commitments for adults, and for children, it can impact their attendance at day care or school which may have educational implications [29, 31, 32].

However, even if a client lives in close proximity to services, there is also the consideration of whether scheduling a clinic-based appointment is in the best interests of the client and their current health state. In some circumstances, it may be better for the client to access care from their own home, avoiding costs, health impact, and travel burden. This issue was highlighted by Collins et al., [33] who examined the feasibility of using telepractice to provide post-discharge supports for adults who had just completed chemo/radiotherapy for HNC. Understanding that these individuals were still unwell from treatment related toxicities, telepractice was used to successfully support post treatment SLP and dietetics reviews into the client’s home [33].

Indeed, the health state of the individual can dramatically increase the effort needed to commute to an in-person clinic appointment, and this has been noted as a particular issue for families traveling to appointments with children with significant physical disabilities and/or specialist medical equipment (e.g., oxygen, suction equipment) [31, 34]. Nicholl [34] interviewed parents of children with complex healthcare needs and identified that parents reported travel outside the home, regardless of distance, required careful preparation and pre-emptive care. Mothers reported that they needed to anticipate sudden or unexpected changes in their child’s condition, and to monitor their child’s health while driving due to previous sudden and unexpected emergencies during travel. It is then also important to recognize the real effects of fatigue associated with travel to the clinic [34] and/or increased levels of anxiety and/or stress associated with attending a healthcare facility that may also negatively impact feeding/swallowing performance [32, 35].

Then, clinicians must also consider the context in which we are assessing the swallowing and mealtime behaviors of our patients, and how representative these assessments are of “usual behavior”. For example, when managing patients with dementia, it is recognized that there are resident, caregiver, and environmental factors which can all influence the success of oral intake [36]. Hence, being able to observe a mealtime conducted within a familiar environment, with familiar foods and feeding assistance, may help provide a more representative picture of an individuals daily functioning than assessing them within an unfamiliar clinic setting. This issue is also particularly relevant in the field of pediatric feeding disorders, where the option of assessing children in their home environment can provide an opportunity to conduct a more naturalistic assessment. While parents are encouraged to bring their child’s usual food, utensils, and equipment to their in-clinic feeding appointments, replicating the child’s usual feeding environment can be difficult to achieve in the clinical setting. In Raatz et al.’s [29] study many parents reported that their child’s feeding behaviors differed between the home and clinic environment. Clinicians have also reported that conducting assessments in the home environment via telepractice improved their clinical decision-making and provided information they would not have obtained in the clinic [37].

In combination, the issues detailed in this section highlight the need for clinicians, organizations, and governing bodies to review their current dysphagia care provision and re-consider if the traditional in-person/in-clinic model should remain as the “only way” services are offered. As the evidence supports, implementing some sessions via telepractice, in combination with in-person care, may help to enhance service efficiencies and reduce travel and financial costs for our clients, many of whom are already experiencing increased stress and care burden. At the very least, all facilities should conduct a regular review of their services and have a clear understanding of the needs of their consumers, to identify how services may better these needs. Given the heterogeneity of our consumers, no one service model or approach can ever meet the needs of all clients. However, establishing integrated services, where a combination of in-person and telepractice modalities are available, and are used when most suited to the clinical task and the clients’ situation, will help to further optimize services for our clients.

## Aspects of Dysphagia Services Can Be Provided via Telepractice

The evidence base to support the use of telepractice to deliver aspects of dysphagia assessment and management continues to emerge. Largely completed prior to the pandemic, studies have been conducted in both adult and pediatric practice areas, and support the use of videoconferencing for conducting components of the clinical assessment (for adults and pediatrics), delivery of some standardized screening tools, and supporting clinical decision-making during videofluoroscopy (VFSS). Providing home-based therapy support for dysphagia management via telepractice has also been successful. The following information is provided as an overview of the current state of the literature and is not intended to be a comprehensive review nor critical analysis of the literature. More detailed reviews of the current evidence and considerations for practice can be found elsewhere [38]. Rather, the intent here is to simply highlight the types of models that have been implemented successfully in dysphagia management, and provide the foundation for the development of future care models.

### Delivering Adult Clinical Swallowing Examinations (CSE) via Telepractice

CSEs conducted via telepractice have been shown to have high levels of interrater reliability with in-person decisions made regarding oromotor function, food and fluid trials, and clinical decision-making and recommendations [39–47]. Although the CSE is not the definitive diagnostic assessment for the presence of dysphagia, it remains a critical component in the assessment process, providing valuable information on the capacity and capability of the patient, functional challenges experienced during food and fluid intake, and insight into dysphagia risk. Outcomes of the CSE are typically used to triage patients into instrumental assessments, and continue to be a method used by clinicians to monitor functional oral intake and progress made during therapy.

The work to develop a model for delivering CSEs via telepractice for the adult population began over a decade ago [44, 45] culminating in a protocol for conducting CSEs via telepractice with adult clients [43]. The clinical model involves the telepractice SLP, as well as an assistant based at the patient end (for patient safety/emergency response and to assist with the session), and a number of strategies to help optimize data collection online (e.g., use of colored fluid/fluids with clear plastic spoons and cups to enhance visualization of the bolus) [43]. Using this basic model, studies have confirmed that patient observations, oromotor assessment items, and food and fluid trials could be conducted with high levels of clinical agreement with in-person assessments [40–42]. Of note, the randomized controlled trial which examined the impacts of dysphagia severity [42] confirmed that patients of all severities were able to be assessed via telepractice. However, clinicians found some sessions conducted with patients with severe dysphagia to be more difficult [42], highlighting the importance for clinicians to develop basic skills and confidence in conducting telepractice assessments prior to undertaking more complex case management online.

Positive evidence for CSE delivery via telepractice has also been confirmed by studies conducted by other research groups, using different assessment tasks, and engaging different clinical populations. In 2017, Morrell et al. [40] examined the validity of telepractice CSEs for acute stroke patients. A bedside nurse assisted the telepractice consult, however, they did not complete any prior additional training contrary to previous studies [41, 42, 47]. Again, excellent agreement (> 85%) was achieved between the online and in-person decisions [40]. Most recently, Borders et al. [39] examined the use of standardized screening tools including the Timed Water Swallow Test (TWST) and the Test of Masticating and Swallowing Solids (TOMASS), as well as clinical observations all conducted via telepractice with a cohort of patients with movement disorders. Assessments were conducted in the patients’ homes, using a range of consumer-grade equipment (e.g., laptop computer). Participants were required to have a carer or family member present at home for the assessment for safety purposes; however, this person did not act in an assistant-role as per prior research. Although ideal visualization criteria was not met across all trials, results still indicated acceptable levels of inter and intra-rater reliability.

Conducting CSEs via telepractice has also been incorporated in models of HNC care [48, 49]. Burns et al. [49] first described using telepractice to provide specialist speech pathology services to patients managed for HNC, where dysphagia was just one component of care delivery. That research was later expanded into a multisite randomized controlled trial which demonstrated improved service efficiency using the telepractice model [48]. Collins et al. [33] used telepractice to provide a home-based telepractice model for the delivery of SLP and dietetics management following HNC treatment. Data revealed the telepractice model of care was more efficient than the traditional in-person model due to a reduction in the number and duration of appointments required [33].

### Use of Telepractice Supported VFSS Assessment

Evidence is also emerging supporting the use of telepractice for improved access to, and expert decision-making for VFSS [50–52]. This concept was initially explored by Malandraki et al. [52], where patients underwent VFSS directed by a clinician located in a research laboratory. The clinical images were transmitted from the radiology suite to the telepractice SLP. At that time, transmission delays and inconsistent image quality influenced the ability to accurately interpret the VFSS images online and the authors highlighted the importance of using high data transfer rates to optimize image quality.

In 2014 Burns and colleagues [53] compared the change in image quality when videofluoroscopic images were transmitted from a digital fluoroscopy system to a) clinical equipment and b) a range of videoconferencing configurations utilizing different network speeds. Findings confirmed that clinical images transmitted via telepractice were equivalent to if not superior to the image quality represented by the in-room imaging system. The outcomes of that study then informed the technical system configuration used in subsequent work examining the feasibility and reliability of conducting real time VFSS via telepractice [50]. Results confirmed high levels of agreement between the online rater and the in-person rater for critical clinical features (i.e., presence of airway invasion/residue, patient response to airway invasion/ residue, effectiveness in clearing airway invasion/residue) and management decisions (i.e., diet/fluid prescription, recommended compensatory strategies, and onward referral to other professionals). This confirmed the potential to conduct remote VFSS via telepractice with a technical system that was configured to optimize the online transfer high quality clinical images between sites.

Telepractice has also been utilized effectively to support clinical decision-making for VFSS. In a study by Malandraki et al. [51] a physician in Greece was trained to conduct and interpret VFSS studies. The recorded images from 17 VFSS assessments conducted in Greece were then transferred to an expert SLP in the USA. Both the clinician in Greece and expert SLP rated the studies based on set diagnostic criteria, dysphagia severity, and management recommendations, and their ratings were compared for agreement. While agreement for diagnostic and severity ratings was adequate, patient management would have been suboptimal for more than half of the patients assessed if review by the expert SLP had not occurred. Hence demonstrating the quality of dysphagia care can be improved and optimized by the use of asynchronous VFSS telepractice models [51].

### Use of Telepractice Within Pediatric Feeding

In the field of pediatric feeding disorders and dysphagia, early work by Clawson et al. [54] described the use of telepractice to deliver specialist multidisciplinary feeding assessments. Children from remote communities participated in a synchronous telepractice appointment that connected a specialist multidisciplinary metropolitan feeding team with families and local care providers. That study confirmed that the telepractice assessment model was feasible, and the model helped families avoid travel and receive services with their local community providers. Rojjanasrirat et al. [55] later investigated the feasibility and interrater reliability of breastfeeding assessments conducted via telepractice by a lactation consultant using the LATCH breastfeeding assessment tool. Again, acceptable levels of interrater reliability were reached for most items assessed during the sessions.

In 2016, Kantarcigil et al. [56] completed a prospective cohort study investigating the validity and reliability of asynchronous telepractice assessments conducted using the Dysphagia Disorders Survey (DDS) and the Dysphagia Management Staging Scale (DMSS) [57]. Nineteen children with cerebral palsy participated in in-person feeding assessments that were video recorded. The video recordings were then reviewed 3 months later by the same or a different SLP using the same assessment tools. High levels of intra- and inter- rater reliability were identified across most variables on the DDS and DMSS [56]. Overall, the study provided positive preliminary support regarding the feasibility and reliability of asynchronous speech pathology assessments conducted via telepractice for children with feeding disorders.

Most recently, work by Raatz et al. [37, 58, 59] developed and then investigated the inter-rater reliability of a system to assess children’s eating, cup drinking, and/or bottle-feeding skills via telepractice. Using a four-phase iterative design the authors developed and piloted the system architecture for conducting synchronous pediatric feeding assessments via telepractice [59]. This system was then used to evaluate the bottle-feeding skills of 30 infants [58] and the eating and/or cup drinking skills of 40 children [37] with pediatric feeding disorders (including dysphagia) at home. Results indicated high levels of agreement for both cohorts; > 85% for all assessment elements except intraoral examination (palate integrity and tonsils) for eating and/or cup drinking assessments [37] and > 80% for all assessment elements except assessment of palate integrity, gagging during non-nutritive suck assessment, and 6/8 components of a tongue tie screen for bottle-feeding assessments [58].

In addition to telepractice assessment validation, studies have also demonstrated the feasibility and clinical applicability of using telepractice for pediatric feeding intervention. In 2014, Malandraki et al. [60] reported positive outcomes following a 4-week telepractice intervention block with one child, which resulted in increased acceptance of food and fluids, increased acceptance of textures, and improved scores on the Eating Assessment Tool (EAT-10) [60]. Marinschek et al. [61] also described a retrospective cohort study investigating the outcomes of tube-weaning delivered using their “net-coaching” (telepractice) model compared to their traditional onsite intensive program. Overall, they found that the incidence of “totally weaned” children was comparable between the two groups.

More recent studies have demonstrated the ability to deliver behavioral feeding intervention for children with avoidant/restrictive food intake disorder (ARFID) [62, 63]. Bloomfield et al. [62] demonstrated that telepractice intervention resulted in increased acceptance of targeted foods and improved parent perceptions of mealtimes. Peterson et al. [63] compared feeding interventions delivered in-person versus via telepractice for a group of 15 children with ARFID. Children and parents completed an intensive day-treatment program prior to participating in their outpatient/telepractice appointments. Findings indicated that children’s behavior was similar for both the in-person and telepractice modalities, and children met an equivalent percentage of their goals across both the appointment conditions. The authors concluded that the telepractice intervention was equally as effective as in-person intervention, though it was acknowledged that participants had received extensive training and instruction prior to the telepractice appointments.

## Telepractice Can Be Used in Flexible Ways to Support Delivery of Dysphagia Services

There are numerous different ways in which telepractice can be incorporated into dysphagia services. As demonstrated by the efficacy studies conducted to date, videoconferencing can be used to connect a client with their clinician to conduct various aspects of dysphagia assessment and management. This connection may be into another service/facility/health setting (i.e., connecting to a smaller hospital setting where SLP services are not available, e.g., Burns et al. [27]), or can be a connection into the patient’s home (e.g., Collins et al. [33], Raatz et al. [37, 58]). Videoconferencing sessions into the home can relieve travel burden, which can be particularly useful in the delivery of intensive therapy programs where in-person clinic visits can be supplemented by some telepractice sessions to minimize the overall travel burden for patients. Sessions in the home also provide more opportunities for naturalistic observation, as well as greater opportunity for engagement with family and carers, who may not otherwise have been able to attend a clinic-based appointment.

The use of videoconferencing to link clients and local care clinicians with experts/other teams has also been shown to be highly beneficial [33, 48, 49, 54]. Using telepractice in this way to support shared-care models between local and specialist care providers is way to address some of the challenges that patients face attending specialist services. Enabling patients to receive care in their local community, supported by a specialist clinician who can join in the sessions via telepractice, can help patients access specialist services closer to home (e.g., Burns et al. [48]). Connecting multiple professionals into a session to deliver interdisciplinary goals is also easily achieved via telepractice. As reported by Collins et al. [33], patients were able to link into a joint session attended by both speech pathology and dietetics, which helped to maximize the efficiency of the patients’ post-discharge appointments.

From our own prior experiences and work in other fields (e.g., [64, 65]), telepractice also provides the potential to offer online group sessions for patients with dysphagia. For example, providing telepractice sessions for clients and their wider families to discuss care, or conducting sessions with groups of patients and carers on modified meal preparation, or using telepractice to present to a group of patients with degenerative conditions about disease impacts to swallowing. Models such as this could provide potential efficiencies for the health service, and also opens up opportunities for the wider family unit to be involved in care. Telepractice group sessions can also be used by consumer groups to establish support groups and networking opportunities for individuals with dysphagia seeking peer support.

For patients with HNC, the emergence of intensive, prophylactic swallowing therapy has also afforded opportunities to expand the use of asynchronous (store-and-forward) telepractice applications for therapeutic purposes to help maximize service access and support intensive treatment models. These custom-built applications use delayed delivery, and store-and-forward of data from one site to another for later review, typically via a mobile/app-based patient portal, with video/audio/text-based instructions for exercises, reminder features, and the ability to log/submit exercise data and communicate with clinicians via messaging and short surveys. Pilot studies [66, 67] have confirmed good engagement and high patient satisfaction with these applications. Subsequent randomized trials have confirmed the ability of these asynchronous telepractice applications to yield equivalent therapy adherence and clinical outcomes to in-person clinician-directed therapy, with superior adherence and patient satisfaction [68, 69]. Novel research is also currently exploring the capabilities of wearable devices (using SEMG and accelerometry) to assist in monitoring dysphagia function and therapy [70–72]. While the use of wearable devices is still in its early inception stage, studies are highlighting a future where applications will assist remote monitoring of swallow function, and will ultimately become part of synchronous and asynchronous telepractice applications.

Asynchronous telepractice has also been applied to promote patient self-monitoring and education. Web-based resources have been designed to educate patients on the treatment trajectory and promote proactive management/coping [66, 73]. Recent investigations have also trialed the use of web-based screening tools for patients during (chemo)radiotherapy treatment, as well as their carers/family members. Such tools incorporate asynchronous store-and-forward features, whereby patients/carers enter data via related to side-effects (including dysphagia and associated sequelae), which are then collated and summarized for the multidisciplinary team to review and use to inform clinical intervention [74, 75].

The potential use of telepractice within a busy dysphagia service also extends beyond direct patient care, with telepractice offering powerful opportunities for clinical training as well as mentoring/supervision. Telepractice offers opportunities for clinicians to link in with more experienced clinicians and learn by observing experts working with clients in other settings. Equally it allows experts to link into sessions to provide mentoring and guidance to clinicians with less experience. Previous research has demonstrated that telesupervision is a feasible and acceptable supervision method [76, 77]. Malandraki et al. [51] used telepractice to provide expert consultation to improve the quality of care for patients with dysphagia following VFSS. Other work by Mayadevi et al. [78] used telepractice to link into specialist multidisciplinary team support to assist treatment planning for patients with complex dysphagia post-HNC, revealing new management decisions, and ultimately improved outcomes for a number of clients through this process. Telepractice may be used more broadly to support “communities of practice”, where clinicians with similar interests and needs can come together online to pool resources and experience peer-based learning and support [79, 80].

## Providing Quality Dysphagia Services via Telepractice Requires Planned Implementation and Ongoing Evaluation

While the increased interest in telepractice created by the COVID pandemic has been positive, there have also been recognized challenges associated with the forced adoption and rapid uptake of telepractice services [9, 18, 81]. There are real concerns that some clinicians and organizations adopted telepractice without the training, infrastructure, and experience that would be expected in less-urgent times, and that this may have impacted the safety, effectiveness, and acceptance of some telepractice models [9, 18, 81]. The lessons learnt during this rapid transition to telepractice require some pause, reflection, and reconsideration. Many of the “failures” experienced can be tracked back to inadequate time for service implementation, and a lack of skills, knowledge training to work effectively in a telepractice environment. For others, it was a lack of access to appropriate resources that created major barriers. Negative clinician perceptions about perceived issues with the “quality” of telepractice services also created initial clinician reluctance and doubt. All of this was not unexpected.

As the field of implementation science has repeatedly shown, the implementation of any new clinical model takes considered time and effort. It also requires an understanding of the internal and external forces that drive change, and a clear understanding of the benefits that service change can bring for consumers and the service itself. Successful implementation also requires staff to clearly see the benefits of the new model. This was demonstrated in a recent study into the experiences of staff from regional/rural services who implemented CSEs via telepractice. Findings revealed that the key drivers of successful implementation were clinicians having a strong sense of the relative advantage of the service model, and knowing staff were well trained and supported to run the service [26].

Then, clinical teams need to have adequate supports to help make this service change happen. Just like establishing any new service, the development of telepractice services must involve careful consideration of numerous factors [16, 18, 82, 83]. It is important for clinicians to have a robust understanding of their clinical service, care requirements, consumer needs, and technology options when developing telepractice services [83]. To help guide clinicians through the complex process of implementation, Martínez-Alcalá et al. [82] outlined that the development of user-centered telepractice services requires four stages: *analysis, design, implementation, and evaluation*, with each of these discussed further here in this section.

### Task Analysis

A *task analysis* is one of the first crucial steps in developing effective telepractice services [82, 83]. This step typically involves clinicians considering the type/s of tasks to be completed via telepractice, the intended end users, and the type of environment/s that will be used [83]. For example, clinicians may consider their appointment goal/s, the clinical tasks they need to complete, typical appointment length, user communication needs, and the type of data to be collected [83]. Clemensen et al. [84] reported that participatory design strategies can be beneficial in this step and they encourage clinicians to actively engage with intended participants to identify user-specific needs and to collaboratively generate ideas and solutions. Work by Raatz et al. [59] is a good example of this process in action, revealing how the tasks required for a pediatric feeding assessment were identified, tested, and trialed using different equipment and set ups to determine suitable options for a service model for pediatric feeding assessments.

### Design

Once the analysis stage has been completed, the *design* phase focuses on the conceptualization of user requirements and the development and trial of the telepractice system. The design process is imperative, as poorly designed systems can negatively impact telepractice uptake, usability, and sustainability [9, 81, 83]. In a recent systematic review, Almathami et al. [85] identified system-design issues, including environmental obstruction, difficulties using the system, technological incompatibility, and even device size and weight, as being barriers to telepractice uptake. Clinicians should be able to identify what type/s of technology would be the most effective for their service and task needs, and how they will adapt tasks for the telepractice environment [59]. It is recommended that clinicians engage in a testing phase where they pilot their developed system with end users to reflect and redesign the system prior to implementation [59, 84].

Integral to this design phase is also consideration of existing policy and infrastructure [85–87]. Technology infrastructure can impact internet access and internet speed in some areas, which can influence the availability and quality of telepractice services [85]. Issues with security and privacy are also frequently cited as barriers to telepractice service delivery [85, 87, 88]. While levels of data security are mandated in some countries (e.g., the USA, Australia), they are not in others [89]. Data security is a serious issue, and providers and patients will continue to lack trust in telepractice services without adequate security and privacy protections. Finally, issues with payment and reimbursement also need to be addressed for telepractice models to be successful and sustainable [85–87].

### Implementation

The *implementation stage* [82] then involves use of the designed telepractice system in clinical care. In this phase, clinicians need to have a strong understanding of when and why to use telepractice, as well as the ability to assess patient suitability, practice readiness, and technology needs [81]. Thomas et al. [9] reported that the COVID-19 pandemic has reinforced that a large proportion of the health workforce have not been adequately trained in how to deliver care via telepractice. They emphasized the need for telepractice to be embedded in university and health training programs to ensure that graduates are “telepractice ready” and discussed the need for the development of discipline-specific guidelines, ongoing staff training, and for professional associations to consider telepractice accreditation [9]. Part of this training is not only having the knowledge of how to deliver a specific task via telepractice, but also an awareness of the larger issues surrounding a clinical telepractice service. For example, Galpin et al. [81] stressed the importance of recognizing patient safety issues when delivering telepractice services. Appropriate education and preparation of patients to ensure they are able to competently and confidently use telepractice is also crucial to implementation success [81]. Furthermore, organizations and individuals must have a comprehensive understanding of their national, state, local, facility, and practice standards, as well as funding requirements, and ensure that they communicate effectively, act professionally, and maintain ethical behavior during telepractice service provision [9, 81].

### Evaluation

Martínez-Alcalá et al. [82] described the final step of the process to be *evaluation*. A comprehensive and ongoing method of evaluation is required to ensure that any new telepractice model is both effective and continues to meet both service and user needs. This evaluation process should be multifactorial and not only examine the effectiveness of telepractice, but also examine clinician and patient experience/satisfaction, patient outcomes (including quality of life), and economic outcomes [9, 84, 90]. It should also try to capture the many traditional benefits of telepractice such as time and financial savings, improved service access, and increased convenience for consumers [27, 30, 33, 54, 85, 91, 92] as well as consider new benefits identified since COVID-19, such as the ability to continue delivery of clinical care during stay-at-home orders [6, 15, 93] and reducing the need for personal protective equipment in times of resource shortages. As there are many potential impacts from introducing telepractice, service evaluations need to be robust, and sensitive to the context in which the service was introduced, in order to fully capture the impact of new telepractice services.

Understanding clinician and client perceptions is another key component of any telepractice service evaluation, as studies have demonstrated that end user satisfaction can significantly influence the uptake and sustainability of telepractice [94]. Variables evaluated in the literature have included comfort, perceived privacy, ease of use, technical functionality, user experience, and perceived usefulness of telepractice [95, 96]. Standardized questionnaires, purpose-built questionnaires, and/or qualitative interviews have all be used to examine perceptions and satisfaction [90, 95, 96].

In particular, understanding clinician attitudes about telepractice is crucial, as it has long been recognized as one of the main barriers impacting uptake of telepractice services [86, 97–99]. A common misperception held by clinicians who have not experienced telepractice, is that telepractice is a “lower quality” service option, offering patients “less” than a traditional in-person service model. However, it has also been shown that once clinicians have had exposure and opportunity to use telepractice, these perceptions change [99]. Indeed, studies of clinician perceptions delivering adult dysphagia telepractice models have revealed high satisfaction, with SLPs reporting that they are able to complete the assessment adequately, establish patient rapport, and use technology easily [27, 33, 41, 47–49]. Such positive clinician feedback has also been found in pediatric telepractice models [37, 54, 58]. Although Ward et al. [47] and Ward et al. [41] did report that in a small number of adult CSE sessions, the telepractice clinicians were not satisfied with the session, further analysis identified that these particular sessions involved patients who were unable to follow verbal instructions, experienced fatigue, distress, or agitation, and had significant hearing or vision impairments or excess body movements [46]. All of these characteristics would create challenges during in-person consultations as well.

Examining client perceptions is also integral when evaluating the success of any telepractice service. Clinicians have long misperceived that certain clients would not be interested in, or accepting of, telepractice models of care, and/or that clients do not have the appropriate computer skills and technology to access these services (particularly for older adults) [97, 98]. However, in direct contrast to these early assumptions, research into consumer perceptions of telepractice models used in both adult [27, 33, 48, 100] and pediatric dysphagia care [37, 54, 58, 60, 62] has demonstrated high levels of consumer satisfaction. From the early work conducted with adult CSE’s delivered via telepractice, it is recognized that a small proportion of patients will still prefer to attend traditional in-person appointments. However, the majority felt that the telepractice appointment was equal to and could indeed replace the traditional in-person consultation [47, 100]. Overall, studies have shown that our clients simply want to be given the choice to consider where telepractice can be part of their own care pathway [16].

Finally, the cost attributed with delivering a clinical service is a key factor impacting sustainability [92] and must be part of the evaluation of any service model. Telepractice services can be examined with a variety of economic analysis methods [101] which may include cost-minimization analysis, cost-effectiveness analysis, cost–benefit analysis, and cost-consequence analysis [92, 102]. In particular, Snoswell et al. [103] emphasized the importance of taking a broader societal perspective when evaluating telepractice models to enable capture of extra clinical costs such as patient-funded travel or loss of productivity.

The cost advantages of telepractice models have been proven in a number of studies. Wade et al.’s [92] systematic review of 36 telepractice models that involved an economic analysis, identified that 61% of studies found their telepractice model of care was less costly than the non-telepractice alternative. They also found that a third of the studies demonstrated improved health outcomes using a telepractice model. Specific to the field of dysphagia services, studies have highlighted cost benefits for models that include the delivery of CSE via telepractice with adult clients [27]. Positive cost benefits have also been reported for telepractice models supporting patients with HNC [33, 104]. There are also positive data for asynchronous therapy models. Wall et al. [105] undertook an economic analysis of a randomized controlled trial comparing the delivery of a prophylactic swallowing therapy program via (1) an asynchronous telepractice app, *“SwallowIT”*, versus (2) clinician-directed in-person therapy and (3) patient self-directed therapy without the app. Findings confirmed that telepractice was the most financially viable model of care, demonstrating higher *cost-efficiency* than in-person therapy (with total cost saving to health service and patients of $1901 AUD per patient). The *SwallowIT* model also proved more *cost-effective* than patient self-directed therapy, yielding clinically significantly superior QoL at the end of treatment, for comparable costs.

Positive cost savings have also been reported regarding with pediatric telepractice feeding services. Clawson et al. [54] identified that their telepractice appointment saved families $899 USD per appointment due to avoidance of travel-related costs, and the telepractice appointment also saved parents 1.5 days away from their usual duties. In Clark et al.’s [106] study, the authors estimated fuel cost savings of $375 USD per family for their 10-appointment series. Similarly, Raatz et al. [30] identified significant time and cost savings (average AUD $95.09 per appointment) associated with their telepractice appointment model for families who lived in close proximity to their feeding service (within a 40 km radius).

## The Future of Telepractice Research

Although research into telepractice models to support dysphagia care began almost 20 years ago, it has been the past decade which has seen the greatest growth in the evidence base for telepractice in dysphagia care. While it is acknowledged that the work to date has helped support many services adopt telepractice, there is a need for more, and a greater consolidation of the research evidence for using telepractice in dysphagia management. Although there are recognized differences in adult and pediatric dysphagia practice, the ongoing agenda for research in both fields is largely the same, and includes further evidence supporting the effectiveness of different models across different clinical settings and client groups; more robust evaluation of the clinical and service outcomes of telepractice models through large scale clinical trials; greater understanding of the clinical application of telepractice through large scale implementation evaluations; further insight into consumer perceptions, including perceptions of system design and usability, and engaging consumers in co-design of clinical telepractice services; and finally, more detailed evidence regarding telepractice service costs and cost-effectiveness.

Given the recommendation for telepractice models to be at least equivalent to traditional in-person services, a primary focus of the ongoing research agenda remains confirming the feasibility, safety, validity, and reliability of telepractice models for dysphagia management. Ongoing work is needed to further refine system designs/technology, and confirm the feasibility of conducting certain clinical assessment and management tasks using technology. The goal to date of most research in this field has been to confirm where telepractice models can achieve “equivalent” outcomes to traditional in-person care. However, it is also possible that some telepractice models may actually be superior to in-person care (e.g., providing dysphagia support for carers providing home palliative care) and these models need full and detailed exploration of the situations and contexts where telepractice may ultimately become best practice. Then once developed and tested in controlled environments, all models need implementation and evaluation in clinical contexts. This data is needed to confirm that the models can achieve the desired outcomes for both patients and services, and help identify factors that influence successful and non-successful implementation.

As previously discussed in this paper, all telepractice models should undergo rigorous exploration of consumer (patient and clinician) satisfaction. However, future research into consumer satisfaction needs to go beyond collecting satisfaction measures [96], and rather explore in greater detail what consumers want, and expect, from telepractice services. This will require engaging with consumers in critical reflection about what they want from their dysphagia services in general, and what they see as “quality” in health care, and then how telepractice models can help meet these ideals. This includes exploring consumer perceptions about the design of systems and usability of technology, and what’s needed to enhance their experience and optimize engagement with their care providers.

Finally, in an era of value-based healthcare, evaluating the costs associated with any new service model, including telepractice, is paramount to determine its true clinical viability and sustainability in routine care. To this end, ensuring that there is systematic research into both the costs, as well as the cost-effectiveness of telepractice models in dysphagia care is a clear direction for future research. Undertaking robust economic analyses in collaboration with health economists will ensure that decision-makers have the necessary evidence to determine the potential benefit of telepractice, and hypothesize changes in cost-effectiveness of service models implemented under different conditions [102].

## Conclusion

Telepractice was proven to be a viable model for providing aspects of dysphagia services long prior to the recent pandemic. While the COVID-19 pandemic has provided the catalyst for more widespread uptake of telepractice in the field of dysphagia care, it is important that this momentum continues, and telepractice is not simply regarded as a “short-term” pandemic solution. It is crucial that the needs of our clients are the central drivers to how we deliver care. Each person and their personal situation must be considered to determine if incorporating telepractice can help them receive quality dysphagia care, in ways that best meet their needs. To this end we must continue to drive research that examines the feasibility, validity, and safety of new telepractice models, and conduct evaluations of wide scale implementations, to build a robust evidence base to support the integration of telepractice into “usual care”. Like all new models of care, clinician training, increased exposure, and experiential learnings will all be integral to ensuring telepractice is used appropriately and delivered in ways that provide quality services. As clinical care continues to move forward into the era of modern health care, it is imperative that the early challenges of the establishing care in an online environment are not allowed to outweigh the benefits.

## References

[1] American Speech-Language-Hearing Association. 2014 SIG 18 Telepractice services survey results. 2014. https://www.asha.org/siteassets/practice-portal/telepractice/sig-18-telepractice-services-survey-results-by-profession.pdf. Accessed 28 Feb 2021

[2] Hill AJ, Miller L (2012). A survey of the clinical use of telehealth in speech-language pathology across Australia. J Clin Pract Speech-Lang Pathol.

[3] Mohan HS, Anjum A, Rao PKS (2017). A survey of telepractice in speech-language pathology and audiology in India. Int J Telerehabil.

[4] Taylor OD. Speech and language screening for children with medical complexity: a comparison of telepractice and in-person methods. 2017.

[5] Raatz M, Ward EC, Marshall J (2020). Telepractice for the delivery of pediatric feeding services: a survey of practice investigating clinician perceptions and current service models in Australia. Dysphagia.

[6] Chadd K, Moyse K, Enderby P (2021). Impact of COVID-19 pandemic on the speech and language therapy profession and their patients. Front Neurol.

[7] Fong R, Tsai CF, Yiu OY (2021). The implementation of telepractice in speech language pathology in Hong Kong during the COVID-19 pandemic. Telemed e-Health.

[8] Mehrotra A, Ray K, Brockmeyer DM (2020). Rapidly converting to “Virtual Practices”: outpatient care in the era of Covid-19. NEJM Catalyst.

[9] Thomas EE, Haydon HM, Mehrotra A (2020). Building on the momentum: sustaining telehealth beyond COVID-19. J Telemed Telecare.

[10] Kraljević JK, Matić A, Dokoza KP (2020). Telepractice as a reaction to the COVID-19 crisis: insights from Croatian SLP settings. Int J Telerehabil.

[11] Kollia B, Tsiamtsiouris J (2021). Influence of the COVID-19 pandemic on telepractice in speech-language pathology. J Prev Interv Community.

[12] Hao Y, Zhang S, Conner A, Lee NY (2021). The evolution of telepractice use during the COVID-19 pandemic: perspectives of pediatric speech-language pathologists. Int J Environ Res Public Health.

[13] Macoir J, Desmarais C, Martel-Sauvageau V, Monetta L (2021). Proactive changes in clinical practice as a result of the COVID-19 pandemic: survey on use of telepractice by Quebec speech-language pathologists. Int J Lang Commun Disord.

[14] Miles A, Connor NP, Desai RV (2021). Dysphagia care across the continuum: a multidisciplinary dysphagia research society taskforce report of service-delivery during the COVID-19 global pandemic. Dysphagia.

[15] Rule DW, Karia MK (2020). Speech-language pathology and audiology care in the COVID-19 era: shared experiences. Perspect ASHA Special Interest Groups.

[16] Cottrell M, Burns CL, Jones A (2021). Sustaining allied health telehealth services beyond the rapid response to COVID-19: learning from patient and staff experiences at a large quaternary hospital. J Telemed Telecare.

[17] Fisk M, Livingstone A, Pit SW (2020). Telehealth in the context of COVID-19: changing perspectives in Australia, the United Kingdom, and the United States. J Med Internet Res.

[18] Thomas EE, Taylor ML, Ward EC (2022). Beyond forced telehealth adoption: a framework to sustain telehealth among allied health services. J Telemed Telecare.

[19] Dawson C, Capewell R, Ellis S (2020). Dysphagia presentation and management following coronavirus disease 2019: an acute care tertiary centre experience. J Laryngol Otol.

[20] Schindler A, Baijens L, Clave P (2021). ESSD commentary on dysphagia management during COVID pandemia. Dysphagia.

[21] Fritz MA, Howell RJ, Brodsky MB (2021). Moving forward with dysphagia care: Implementing strategies during the COVID-19 pandemic and beyond. Dysphagia.

[22] Namasivayam-MacDonald A, Riquelme L (2020). Speech-language pathology management for adults with COVID-19 in the acute hospital setting: initial recommendations to guide clinical practice. Am J Speech Lang Pathol.

[23] Fong R, Tsai KCF, Tong MCF, Lee KYS (2020). Management of dysphagia in nursing homes during the COVID-19 pandemic: strategies and experiences. SN Compr Clin Med.

[24] Blumenfeld L, Evangelista L, Kuhn M (2020). Management of patients undergoing radiation treatment for head and neck cancer during the COVID-19 pandemic: clinical guidelines and perspectives. Perspect ASHA Special Interest Groups.

[25] Ku PKM, Holsinger FC, Chan JYK (2020). Management of dysphagia in the patient with head and neck cancer during COVID-19 pandemic: practical strategy. Head Neck.

[26] Ward EC, Burns CL, Gray A (2021). Establishing clinical swallowing assessment services via telepractice: a multisite implementation evaluation. Am J Speech Lang Pathol.

[27] Burns CL, Ward EC, Gray A (2019). Implementation of speech pathology telepractice services for clinical swallowing assessment: an evaluation of service outcomes, costs and consumer satisfaction. J Telemed Telecareelemed Telecare.

[28] Burgess A, Purdy S, Jackson B (2016). Allied health professionals’ perspectives of working with dysphagia in a rural paediatric team. N Z Med J.

[29] Raatz M, Ward EC, Marshall J (2021). “It takes a whole day, even though it’s a one-hour appointment!”: factors impacting access to pediatric feeding services. Dysphagia.

[30] Raatz M, Ward EC, Marshall J (2021). A time and cost analysis of speech pathology paediatric feeding services delivered in-person versus via telepractice. J Telemed Telecare.

[31] Ballantyne M, Liscumb L, Brandon E (2019). Mothers’ perceived barriers to and recommendations for health care appointment keeping for children who have cerebral palsy. Global Qual Nurs Res.

[32] Fairweather GC, Lincoln MA, Ramsden R (2016). Speech-language pathology teletherapy in rural and remote educational settings: decreasing service inequities. Int J Speech Lang Pathol.

[33] Collins A, Burns CL, Ward EC (2017). Home-based telehealth service for swallowing and nutritional management following head and neck cancer treatment. J Telemed Telecare.

[34] Nicholl H (2015). ‘Going between worlds’: travelling with children with complex needs. J Child Health Care.

[35] Resnick R, Hergenroeder E (1975). Children and the emergency room. Child Today.

[36] Liu W, Jao YL, Williams K (2019). Factors influencing the pace of food intake for nursing home residents with dementia: resident characteristics, staff mealtime assistance and environmental stimulation. Nurs Open.

[37] Raatz M, Ward EC, Marshall J, Burns CL (2021). Evaluating the use of telepractice to deliver pediatric feeding assessments. Am J Speech-Lang Pathol.

[38] Malandraki GA, Arkenberg RH, Mitchell SS, Malandraki JB (2021). Telehealth for dysphagia across the life span: using contemporary evidence and expertise to guide clinical practice during and after COVID-19. Am J Speech Lang Pathol.

[39] Borders JC, Sevitz JS, Malandraki JB (2021). Objective and subjective clinical swallowing outcomes via telehealth: reliability in outpatient clinical practice. Am J Speech Lang Pathol.

[40] Morrell K, Hyers M, Stuchiner T (2017). Telehealth stroke dysphagia evaluation is safe and effective. Cerebrovasc Dis.

[41] Ward EC, Sharma S, Burns CL (2012). Validity of conducting clinical dysphagia assessments for patients with normal to mild cognitive impairment via telerehabilitation. Dysphagia.

[42] Ward EC, Burns CL, Theodoros DG, Russell TG (2014). Impact of dysphagia severity on clinical decision making via telerehabilitation. Telemed e-Health.

[43] Sharma S, Ward EC, Burns CL (2011). Assessing swallowing disorders online: a pilot telerehabilitation study. Telemed e-Health.

[44] Ward EC, Crombie J, Trickey M (2009). Assessment of communication and swallowing post-laryngectomy: a telerehabilitation trial. J Telemed Telecare.

[45] Ward EC, White J, Russell T (2007). Assessment of communication and swallowing post-laryngectomy: a telerehabilitation trial. J Telemed Telecare.

[46] Ward EC, Sharma S, Burns CL (2012). Managing patient factors in the assessment of swallowing via telerehabilitation. Int J Telemed Appl.

[47] Ward EC, Burns CL, Theodoros DG, Russell TG (2013). Evaluation of a clinical service model for dysphagia assessment via telerehabilitation. Int J Telemed Appl.

[48] Burns CL, Ward EC, Hill AJ (2017). Randomized controlled trial of a multisite speech pathology telepractice service providing swallowing and communication intervention to patients with head and neck cancer: evaluation of service outcomes. Head Neck.

[49] Burns CL, Ward EC, Hill AJ (2012). A pilot trial of a speech pathology telehealth service for head and neck cancer patients. J Telemed Telecare.

[50] Burns CL, Ward EC, Hill AJ (2016). Conducting real-time videofluoroscopic swallow study via telepractice: a preliminary feasibility and reliability study. Dysphagia.

[51] Malandraki GA, Markaki V, Georgopoulos VC (2013). An international pilot study of asynchronous teleconsultation for oropharyngeal dysphagia. J Telemed Telecare.

[52] Malandraki GA, McCullough G, He X (2011). Teledynamic evaluation of oropharyngeal swallowing. J Speech Lang Hear Res.

[53] Burns CL, Ward EC, Hill AJ (2014). The impact of image transfer on image quality in videofluoroscopic swallow studies. Dysphagia.

[54] Clawson B, Selden M, Lacks M (2008). Complex pediatric feeding disorders: using teleconferencing technology to improve access to a treatment program. Pediatr Nurs.

[55] Rojjanasrirat W, Nelson EL, Wambach KA (2012). A pilot study of home-based videoconferencing for breastfeeding support. J Hum Lact.

[56] Kantarcigil C, Sheppard JJ, Gordon AM (2016). A telehealth approach to conducting clinical swallowing evaluations in children with cerebral palsy. Res Dev Disabil.

[57] Sheppard JJ, Hochman R, Baer C (2014). The dysphagia disorder survey: validation of an assessment for swallowing and feeding function in developmental disability. Res Dev Disabil.

[58] Raatz M, Ward EC, Marshall J, Burns CL (2021). Evaluating the use of telepractice for bottle-feeding assessments. Children.

[59] Raatz M, Ward EC, Marshall J, Burns CL (2019). Developing the system architecture for conducting synchronous paediatric feeding assessments via telepractice. J Telemed Telecare.

[60] Malandraki GA, Roth M, Sheppard JJ (2014). Telepractice for pediatric dysphagia: a case study. Int J Telerehabil.

[61] Marinschek S, Dunitz-Scheer M, Pahsini K (2014). Weaning children off enteral nutrition by netcoaching versus onsite treatment: a comparative study. J Paediatr Child Health.

[62] Bloomfield BS, Fischer AJ, Clark RR, Dove MB (2018). Treatment of food selectivity in a child with avoidant/restrictive food intake disorder through parent teleconsultation. Behav Anal Pract.

[63] Peterson KM, Ibañez VF, Volkert VM (2021). Using telehealth to provide outpatient follow-up to children with avoidant/restrictive food intake disorder. J Appl Behav Anal.

[64] Scriven H, Doherty DP, Ward EC (2019). Evaluation of a multisite telehealth group model for persistent pain management for rural/remote participants. Rural Remote Health.

[65] Quinn R, Park S, Theodoros D, Hill AJ (2019). Delivering group speech maintenance therapy via telerehabilitation to people with Parkinson’s disease: a pilot study. Int J Speech Lang Pathol.

[66] Starmer HM, Abrams R, Webster K (2018). Feasibility of a mobile application to enhance swallowing therapy for patients undergoing radiation-based treatment for head and neck cancer. Dysphagia.

[67] Wall LR, Ward EC, Cartmill B (2017). Examining user perceptions of SwallowIT: a pilot study of a new telepractice application for delivering intensive swallowing therapy to head and neck cancer patients. J Telemed Telecare.

[68] Wall LR, Ward EC, Cartmill B (2017). Adherence to a prophylactic swallowing therapy program during (chemo) radiotherapy: impact of service-delivery model and patient factors. Dysphagia.

[69] Wall LR, Ward EC, Cartmill B (2020). Prophylactic swallowing therapy for patients with head and neck cancer: a three-arm randomized parallel-group trial. Head Neck.

[70] Steele CM, Mukherjee R, Kortelainen JM (2019). Development of a non-invasive device for swallow screening in patients at risk of oropharyngeal dysphagia: results from a prospective exploratory study. Dysphagia.

[71] Constantinescu G, Kuffel K, Aalto D (2018). Evaluation of an automated swallow-detection algorithm using visual biofeedback in healthy adults and head and neck cancer survivors. Dysphagia.

[72] Lee Y, Nicholls B, Sup Lee D (2017). Soft electronics enabled ergonomic human-computer interaction for swallowing training. Sci Rep.

[73] Fang CY, Galloway TJ, Egleston BL (2020). Development of a web-based supportive care program for patients with head and neck cancer. Front Oncol.

[74] Wall LR, Cartmill B, Ward EC (2016). “ScreenIT”: computerized screening of swallowing, nutrition and distress in head and neck cancer patients during (chemo)radiotherapy. Oral Oncol.

[75] Wishart LR, Brown B, Nund RL (2021). A prospective study monitoring carer distress during (chemo)radiotherapy for head and neck cancer via an electronic platform. J Med Radiat Sci.

[76] Martin P, Lizarondo L, Kumar S (2018). A systematic review of the factors that influence the quality and effectiveness of telesupervision for health professionals. J Telemed Telecare.

[77] Moran AM, Coyle J, Pope R (2014). Supervision, support and mentoring interventions for health practitioners in rural and remote contexts: An integrative review and thematic synthesis of the literature to identify mechanisms for successful outcomes. Hum Resour Health.

[78] Mayadevi M, Thankappan K, Limbachiya SV (2018). Interdisciplinary telemedicine in the management of dysphagia in head and neck. Dysphagia.

[79] Ranmuthugala G, Plumb JJ, Cunningham FC (2011). How and why are communities of practice established in the healthcare sector? A systematic review of the literature. BMC Health Serv Res.

[80] Seibert S (2015). The meaning of a healthcare community of practice. Nurs Forum.

[81] Galpin K, Sikka N, King SL (2021). Expert consensus: telehealth skills for health care professionals. Telemed e-Health.

[82] Martínez-Alcalá CI, Muñoz M, Monguet-Fierro J (2013). Design and customization of telemedicine systems. Comput Math Methods Med.

[83] Pramuka M, van Roosmalen L (2009). Telerehabilitation technologies: accessibility and usability. Int J Telerehabil.

[84] Clemensen J, Rothmann MJ, Smith AC (2017). Participatory design methods in telemedicine research. J Telemed Telecare.

[85] Almathami HKY, Than Win K, Vlahu-Gjorgievska E (2020). Barriers and facilitators that influence telemedicine-based, real-time, online consultation at patients’ homes: systematic literature review. J Med Internet Res.

[86] Moffatt JJ, Eley DS (2011). Barriers to the up-take of telemedicine in Australia—a view from providers. Rural Remote Health.

[87] Scott Kruse C, Karem P, Shifflett K (2018). Evaluating barriers to adopting telemedicine worldwide: a systematic review. J Telemed Telecare.

[88] Hall JL, Mcgraw D (2014). For telehealth to succeed, privacy and security risks must be identified and addressed. Health Aff.

[89] Department of Health and Human Services (2004). Standards for Privacy of Individually Identifiable Health Information (HIPAA).

[90] Kidholm K, Clemensen J, Caffery LJ, Smith AC (2017). The Model for Assessment of Telemedicine (MAST): a scoping review of empirical studies. J Telemed Telecare.

[91] Molini-Avejonas DR, Rondon-Melo S, de La Higuera Amato CA, Samelli AG (2015). A systematic review of the use of telehealth in speech, language and hearing sciences. J Telemed Telecare.

[92] Wade VA, Karnon J, Elshaug AG, Hiller JE (2010). A systematic review of economic analyses of telehealth services using real time video communication. BMC Health Serv Res.

[93] Tohidast SA, Mansuri B, Bagheri R, Azimi H (2020). Provision of speech-language pathology services for the treatment of speech and language disorders in children during the COVID-19 pandemic: problems, concerns, and solutions. Int J Pediatr Otorhinolaryngol.

[94] Wade VA, Eliott JA, Hiller JE (2014). Clinician acceptance is the key factor for sustainable telehealth services. Qual Health Res.

[95] AlDossary S, Martin-Khan MG, Bradford NK, Smith AC (2017). A systematic review of the methodologies used to evaluate telemedicine service initiatives in hospital facilities. Int J Med Informatics.

[96] Langbecker D, Caffery LJ, Gillespie N, Smith AC (2017). Using survey methods in telehealth research: a practical guide. J Telemed Telecare.

[97] Dunkley C, Pattie L, Wilson L, McAllister L (2010). A comparison of rural speech-language pathologists’ and residents’ access to and attitudes towards the use of technology for speech-language pathology service delivery. Int J Speech Lang Pathol.

[98] May J, Erickson S (2014). Telehealth: why not? Perspectives of speech-language pathologists not engaging in telehealth. J Clin Pract Speech-Lang Pathol.

[99] Hines M, Lincoln M, Ramsden R (2015). Speech pathologists’ perspectives on transitioning to telepractice: what factors promote acceptance?. J Telemed Telecare.

[100] Sharma S, Ward EC, Burns CL (2013). Assessing dysphagia via telerehabilitation: patient perceptions and satisfaction. Int J Speech Lang Pathol.

[101] Goodacre S, McCabe C (2002). An introduction to economic evaluation. Emerg Med J.

[102] Burns CL, Wishart LR, Kularatna S, Ward EC (2020). Knowing the costs of change: an introduction to health economic analyses and considerations for their use in implementation research. Speech Lang Hear.

[103] Snoswell C, Smith AC, Scuffham PA, Whitty JA (2017). Economic evaluation strategies in telehealth: obtaining a more holistic valuation of telehealth interventions. J Telemed Telecare.

[104] Burns CL, Kularatna S, Ward EC (2017). Cost analysis of a speech pathology synchronous telepractice service for patients with head and neck cancer. Head Neck.

[105] Wall LR, Kularatna S, Ward EC (2019). Economic analysis of a three-arm RCT exploring the delivery of intensive, prophylactic swallowing therapy to patients with head and neck cancer during (chemo)radiotherapy. Dysphagia.

[106] Clark RR, Fischer AJ, Lehman EL, Bloomfield BS (2019). Developing and implementing a telehealth enhanced interdisciplinary pediatric feeding disorders clinic: a program description and evaluation. J Dev Phys Disabil.

